# Spatial variation of vector vortex beams with plasmonic metasurfaces

**DOI:** 10.1038/s41598-019-46433-z

**Published:** 2019-07-10

**Authors:** Yuchao Zhang, Jie Gao, Xiaodong Yang

**Affiliations:** Department of Mechanical and Aerospace Engineering, Missouri University of Science and Technology, Rolla, MO 65409 USA

**Keywords:** Nanophotonics and plasmonics, Metamaterials

## Abstract

The spatial variation of vector vortex beams with arbitrary polarization states and orbital angular momentum (OAM) values along the beam propagation is demonstrated by using plasmonic metasurfaces with the initial geometric phase profiles determined from the caustic theory. The vector vortex beam is produced by the superposition of deflected right- and left-handed circularly polarized component vortices with different helical phase charges, which are simultaneously generated off-axially by the single metasurface. Besides, the detailed evolution processes of intensity profile, polarization distribution and OAM value along the beam propagation distance is analyzed. The demonstrated arbitrary space-variant vector vortex beam will pave the way to many promising applications related to spin-to-orbital angular momentum conversion, spin-orbit hybrid entanglement, particle manipulation and transportation, and optical communication.

## Introduction

Optical vector vortex beams have polarization singularities and also possess phase singularities in both their right- and left-handed circularly polarized (RCP and LCP) component vortices. With particular characteristics in polarization and phase structures, vector vortex beams have made a wide variety of applications in the fields of optical tight focus^1–3^, optical tweezing^4–6^, spin-to-orbital angular momentum conversion^7–10^, confocal microscopy^11,12^, and metrology^13,14^. An optical vortex beam with homogeneous polarization state has a helical phase front with azimuthal phase dependency of exp(*ilφ*), where *φ* is the azimuthal angle, *l* is the topological charge (TC) of helical phase with *lħ* representing the OAM carried by each photon. A vector vortex beam with space-variant polarization state has the singularity of polarization which is coincident with the corresponding singularity of helical phase. As the most common vector vortex beam, radially or azimuthally polarized beam is produced by the equally-weighted superposition of two component vortices with opposite circular polarization states and opposite OAM values of *l* = +1 and *l* = −1. While the high-order vector vortex beam is composed by two component vortices with large OAM values and opposite circular polarizations^15^, which can be used for ultrasensitive angular measurement and spin object detection^13,14^. The OAM value and polarization distribution of an optical beam can be changed along the beam propagation in free space. Recently, the topological charge inversion of optical vortex has been demonstrated^16,17^ and the Poincaré beams with polarization transformation during the free space propagation are shown^18^. The vector vortex beams with both varying OAM and polarization state has also been studied^19,20^. It is essential to generate vector vortex beams with arbitrarily polarization states and OAM values varying along the beam propagation, which has not been demonstrated yet.

The conventional method for realizing the spatial variation of vector vortex beams requires bulky free-space optical components including spatial light modulator and Fourier transform lens, which increases the optical system complexity and also limits the photonic integration. Recently, metasurfaces made of nanoantenna arrays in ultrathin metallic films have been provided a powerful platform for tailoring the phase front. By introducing the geometric phase accompanied with polarization conversion^21–26^, metasurfaces have been widely used for building on-chip wavefront shaping devices such as optical vortex generators^27–30^, flat optical lenses^31–36^, thin wave plates^37–40^ and holograms^41–45^.

Here, an effective approach to realize the spatial variation of vector vortex beams with arbitrary polarization states and OAM values of RCP and LCP component vortices along the beam propagation is demonstrated based on the plasmonic metasurfaces with the initial phase profiles designed from the caustic theory. With only a single metasurface, both the deflected RCP and LCP component vortices with different helical phase charges are generated simultaneously, and the vector vortex beam is produced by the superposition of these two component vortices. Furthermore, the detailed evolution processes of intensity profile, polarization distribution and OAM value along the beam propagation distance is presented. The spatial variation of vector vortex beams could have miscellaneous applications due to the polarization singularities and the OAMs carried in both RCP and LCP component vortices. The variation of vector vortex provides different OAMs and polarization states at different propagation distances, which give more degrees of freedom for the applications involved with both OAM and polarization, especially in the regimes of particle manipulation, optical communication and quantum information processing. For the particle manipulation, both OAM and polarization of light can be transferred to microparticles with torques, and different OAMs or polarization states will create different mechanical torques to microparticles. Thereby, the space-variant vector vortex beam could generate space-depended torque that has distinct magnitude and orientation at each location, and such feature could be used as particle and DNA investigations^46,47^. For the optical communication and quantum information processing, both OAM and polarization state can represent the bit and qubit of information. The space-variant OAM and polarization state provide extended degrees of freedom for the entanglement based on both spin and orbital angular momentum^48,49^.

## Results

### Design of plasmonic metasurface

**Figure 1:**
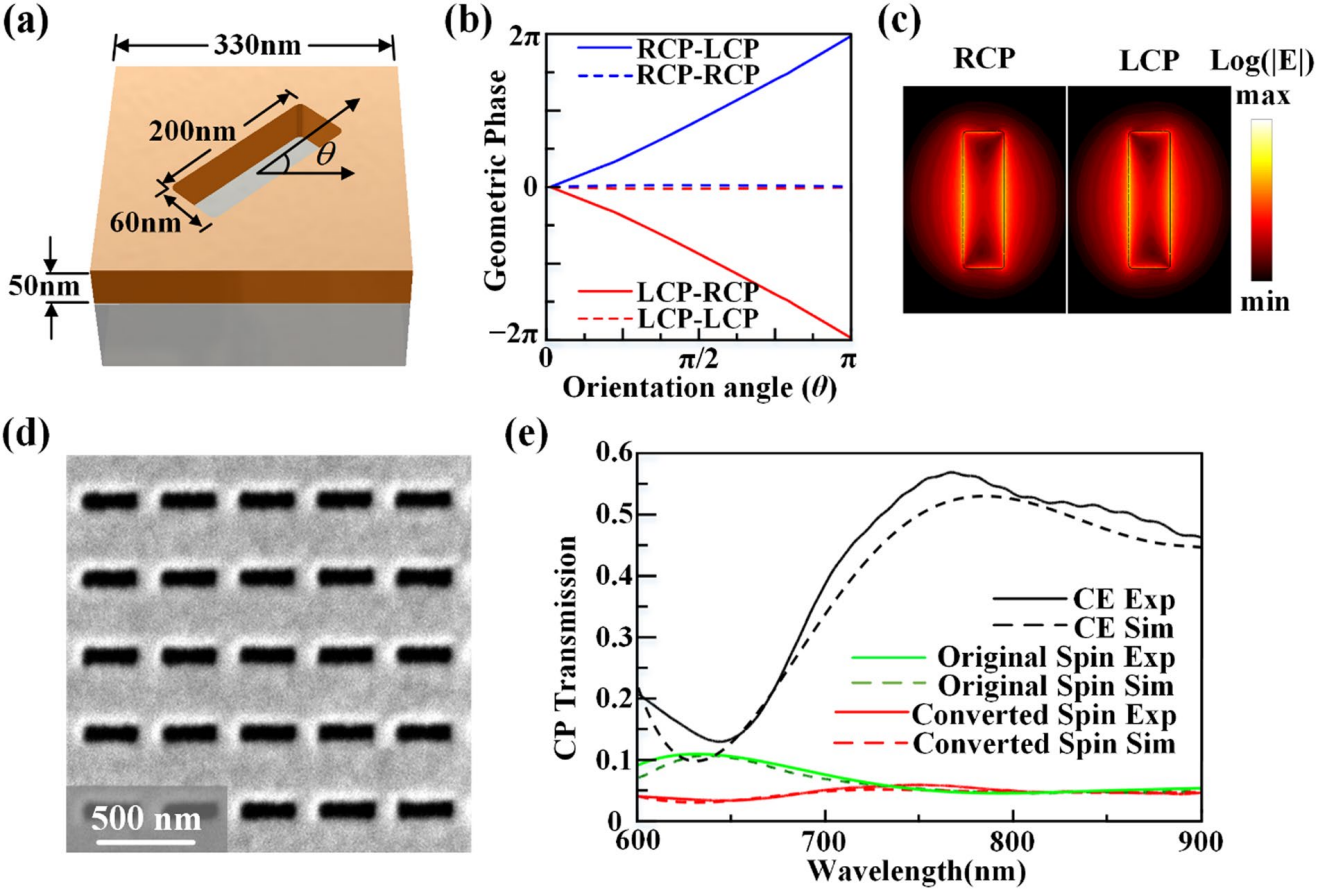
(**a**) Schematic of the unit cell with the nanoslit antenna at orientation angle *θ*. (**b**) Simulated relationship between the phase shift of the converted polarization and the orientation angle of nanoslit antenna. (**c**) Simulated electric field |*E*| distributions of nanoslit antenna under circular polarizations at 633 nm. (**d**) A SEM image of the homogeneous nanoslit antenna array. (**e**) Measured and simulated transmission spectra and conversion efficiency spectra under circular polarization basis.

As shown in Fig. 1(a), the subwavelength nanoslit antennas with different orientation angles are etched in a gold film with thickness of 50 nm on glass substrate using focused ion beam (FIB, FEI Helios Nanolab 600 Dual Beam system). The unit cell of the nanoslit antenna has the period of 330 nm, and the width and length of nanoslit antenna is 60 nm and 200 nm, respectively. As a circularly polarized beam is incident on the metasurface, the introduced geometric phase shift to the converted spin component transmitted from each nanoslit antenna is 2*θ* which is twice as the orientation angle of nanoslit, while the original spin component has no phase shift. Also for RCP incidence the transmitted LCP component has a phase shift of 2*θ*, while for LCP incidence the transmitted RCP component has a reversed phase shift of −2*θ*^15,50^. The simulated relationship between the phase shift of the converted polarization and the orientation angle of nanoslit antenna is shown in Fig. 1(b). Figure 1(c) shows the simulated electric field |E| distributions at the wavelength of 633 nm, where the RCP and LCP components have different field profiles, indicating strong polarization anisotropy. Figure 1(d) gives the SEM image of the fabricated homogeneous array of nanoslit antennas. For the transmission under circular polarization basis, the total transmitted beam through the metasurface contains both the original spin component and the converted spin component. The polarization conversion efficiency is defined as the intensity ratio between the converted spin component and the total transmitted beam. Figure 1(e) plots the measured and simulated spectra of original spin transmission (green lines), converted spin transmission (red lines) and conversion efficiency (CE, black lines). The observed maximum conversion efficiency is around 55% near 800 nm, where the plasmonic resonance occurs. Here a HeNe laser at 633 nm is used in experiments due to its high-quality laser beam for the measurements of both beam intensity profiles and interference patterns. The transmission efficiency of plasmonic metasurfaces is relatively low due to the large Ohmic loss of metal. However, the principle of geometric phase-based metasurfaces can be extended to the recently developed dielectric metasurfaces made of silicon or titanium oxide which have very low absorption loss for manipulating light with ultrahigh transmission efficiency and high polarization conversion efficiency^24–26^.

**Figure 2:**
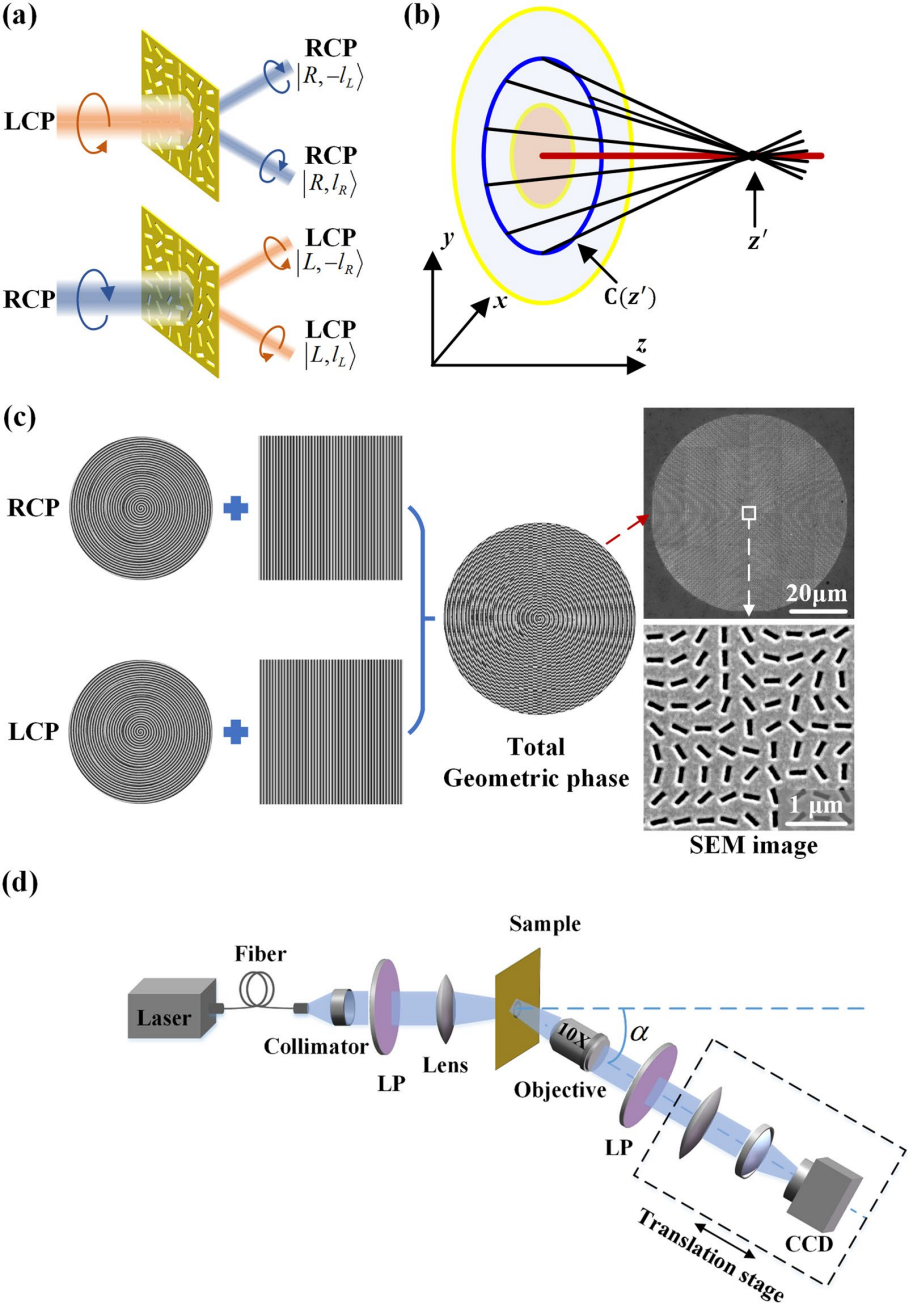
(**a**) The deflected RCP and LCP beams through the metasurface. (**b**) Schematic of the formation of caustic curve. (**c**) Generation steps of the total geometric phase profile for producing the space-variant vector vortex beam. The right column is the SEM images of the whole metasurface (upper figure) and the central part of metasurface marked by the white square (bottom figure). (**d**) Schematic illustration of the experimental setup to characterize the vector vortex beam.

It has been shown that the vector vortex beam can be generated by the superposition of two component vortices with opposite circular polarizations and different helical phase charges, with the optical field for vector vortex beam expressed as^15,51^:

1 $${\boldsymbol{{\rm E}}}=\frac{1}{\sqrt{2}}(|R,\,{l}_{R}\rangle +|L,\,{l}_{L}\rangle )$$

where $|R,\,{l}_{R}\rangle =\exp [i({l}_{R}\phi +{\rm{\Theta }})]{(1,-i)}^{T}$ represents the RCP component with helical phase charge of *l*_R_, $|L,\,{l}_{L}\rangle =\exp \,[i({l}_{L}\phi -{\rm{\Theta }})]{(1,i)}^{T}$ represents the LCP component with charge of *l*_L_, and Θ is a phase constant to represent the phase difference between the RCP and LCP components, so that the total vector field is written as^52^:

2 $$\begin{array}{c}{\boldsymbol{{\rm E}}}=\frac{1}{\sqrt{2}}[\exp [i({l}_{R}\phi +{\rm{\Theta }})](\begin{array}{c}1\\ -i\end{array})+\exp [i({l}_{L}\phi -{\rm{\Theta }})](\begin{array}{c}1\\ i\end{array})]\\ \,=\,\frac{1}{\sqrt{2}}\exp (in\phi )(\begin{array}{c}\cos (m\phi +{\rm{\Theta }})\\ \sin (m\phi +{\rm{\Theta }})\end{array})\end{array}$$

where *m* = (*l*_*R*_ − *l*_*L*_)/2 represents the TC of polarization state for the vector vortex beam, Θ is the initial polarization orientation for *φ* = 0, and *n* = (*l*_*R*_ + *l*_*L*_)/2 is the average OAM value of RCP and LCP component vortices. The parameter *m* describes polarization rotations per round trip along the azimuthal direction, which is called as the index of the vector vortex beam^53^. For example, the case for *n* = 0, *m* = 1 and Θ = 0 corresponds to the radially polarized beam which is a transverse magnetic (TM) beam, while the case for *n* = 0, *m* = 1 and Θ = π/2 gives the azimuthally polarized beam which is a transverse electric (TE) beam. To distinguish the original spin component and the converted spin component, a linear phase gradient is imposed into the geometric phase profile, so that the converted spin component is deflected by an angle with respect to the original spin component. As shown in Fig. 2(a), after passing through the metasurface, the incident LCP or RCP beam is converted into two deflected RCP or LCP vortex beams with the desired helical phase charges. As the incident beam is linearly polarized, the converted beam will be a vector vortex beam with the superposition of both the output RCP and LCP components.

The initial geometric phase profile encoded on the metasurface is determined from the caustic theory. It has been shown that the caustic curve can be generated by modulating the phase on the initial input plane, which has been used to produce a series of nondiffracting Bessel-like beams with arbitrary trajectories^54^. Assuming the initial input field is ${\rm{\Psi }}({x}_{0},\,{y}_{0},z=0)=G({r}_{0})\,\exp (ikQ({x}_{0},{y}_{0}))$, the optical field at propagation distance *z* could be calculated by the paraxial Fresnel integral:

3 $${\rm{\Psi }}(x,y,z)=\frac{\exp (ikz)}{i\lambda z}\int \int G({r}_{0})\exp \{ik[Q({x}_{0},{y}_{0})+\frac{{(x-{x}_{0})}^{2}+{(y-{y}_{0})}^{2}}{2z}]\}d{x}_{0}d{y}_{0}.$$

By applying the stationary phase approach, the optical field is determined by the critical points satisfied the conditions of $(x-{x}_{0})/z=dQ/d{x}_{0}$ and $(y-{y}_{0})/z=dQ/d{y}_{0}$. According to the caustic method^54^, the point at the propagation distance *z* = *z*′ corresponds to a circle *C*(*z* = *z*′) on the input plane and a conical ray bundle emitted from this circle will intersect at this point, as shown in Fig. 2(b). As the circle *C*(*z*) shifts and expands on the input plane, the continuous caustic curve represented by $[f(z),\,g(z),\,z]$ is formed by the locus of the apexes of the conical ray bundles emitted from the circle *C*(*z*). The function of circle *C*(*z*) for generating a conical ray bundle satisfies ${({x}_{0}-{x}_{c})}^{2}+{({y}_{0}-{y}_{c})}^{2}=R{(z)}^{2}$, where $({x}_{c},{y}_{c})$ is the coordinates of the circle center with ${x}_{c}=f-zf^{\prime}$ and ${y}_{c}=g-zg^{\prime}$ and *R*(*z*) is the circle radius. The circle function *C*(*z*) is related to the propagation distance *z* with $z=z({x}_{0},{y}_{0})$. If no vortex is imposed on the initial field, the conical ray bundle emitted from the circle *C*(*z*) is focused at the point $[f(z),\,g(z),\,z]$. In this case, the initial function $Q({x}_{0},{y}_{0})$ is expressed by the formula^54^:

4 $$Q({x}_{0},{y}_{0})=\frac{{\rm{1}}}{2}{\int }_{0}^{z}\{{[f^{\prime} (\xi )]}^{2}+{[g^{\prime} (\xi )]}^{2}-{[\frac{R(\xi )}{\xi }]}^{2}\}d\xi -\frac{{(f-{x}_{0})}^{2}+{(g-{y}_{0})}^{2}}{2z}$$

By adding the vortex phase exp(*ilφ*), the initial input phase is changed into exp[*i*(*kQ* + *lφ*)]. Then the corresponding stationary phase condition becomes $(l/k)\cdot d\phi /d{x}_{0}+dQ/d{x}_{0}=(x-{x}_{0})/z$ and $(l/k)\cdot d\phi /d{y}_{0}+$ $dQ/d{y}_{0}=(y-{y}_{0})/z$. Due to the large wavenumber *k*, the terms (*l/k*)∙*dφ*/*dx*_0_ and (*l/k*)*∙dφ*/*dy*_0_ can be neglected, so that after adding the vortex structure, the optical field at *z* is still majorly contributed by the circle *C*(*z*). In this case, the helical phase on the circle *C*(*z*) is exp(*jlφ*), and by using polar coordinates of $({x}_{0},{y}_{0})={r}_{0}(\cos \,\phi ,\,\sin \,\phi )$ and $(x,y)=\rho (\cos \,\theta ,\,\sin \,\theta )$, the complex field distribution at the propagation distance *z* is calculated by $F(z)=\int \exp (il\phi )\exp (\,-\,ik{r}_{0}\rho \,\cos (\phi -\theta )/z)\,d\phi =2\pi {J}_{l}(k{r}_{0}\rho /z)\exp (il\theta )$^54^, showing a vortex field with charge of *l* and the Bessel-like intensity distribution with order *l*.

In order to generate space-variant vector vortex beam, the initial input plane is divided into four different zones, and different zones have different TCs of helical phase. Since the optical field distribution at the propagation distance *z* is mainly determined by the circle *C*(*z*) on the input plane, vortex fields with helical phase charges varying as the propagation distance can be created by different circular zones with the corresponding charges on the input plane. As the beam propagates, different circular zones will generate optical vortices with varying charges. The yellow dashed circles divide the input plane into different circular zones, and the distance *Zt*(*s*) corresponding to each yellow dashed circle is defined as the transition distance. A function *L*(z) is used to represent the TCs of helical phase at different distances:

5 $$L(z)=\{{l}^{s}|Zt(s-1) < z < Zt(s)\},\,s=1,\,2,\,3,\,4.$$

where *Zt*(*s*) = 20∙*s* (µm). In the current case, the beam propagates along the straight line, so that the parameter function of caustic curve is [*f*(*z*) = 0, *g*(*z*) = 0, *z*] and the radius function is *R*(*z*) = 0.625*z*. The circle function *C*(*z*) is ${x}_{0}^{2}+{y}_{0}^{2}=0.39{z}^{2}$, and thus $z({x}_{0},{y}_{0})=0.625\sqrt{{x}_{0}^{2}+{y}_{0}^{2}}$. The function $Q({x}_{0},{y}_{0})=-\,0.625\sqrt{({x}_{0}^{2}+{y}_{0}^{2})}$ is obtained with Eq. (4). It can be seen that in this case the function $Q({x}_{0},{y}_{0})$ is a simple axicon phase profile. The caustic phase function to realize the space-variant vortex is acquired by adding the helical phase structure $L[z({x}_{0},{y}_{0})]\,\cdot \,\phi$ into $kQ({x}_{0},{y}_{0})$:

6 $$P({x}_{0},{y}_{0})=kQ({x}_{0},{y}_{0})+L(z)\arctan ({y}_{0}/{x}_{0}).$$

The vector vortex beam is produced by the superposition of two vortex beams with opposite circular polarizations and different helical phase charges. Since the metasurface formed by rotating the nanoslit antenna will generate geometric phase profiles of *φ*_*geom*_(*x*, *y*) and −*φ*_*geom*_(*x*, *y*) for the LCP and RCP components, the phase profiles for the LCP and RCP components need to be designed separately. First, for the LCP component, the caustic phase function of Eq. (6) is ${P}_{L}=kQ+{L}_{L}(z)\phi$, where *L*_*L*_(*z*) = $\{{l}_{L}^{1},\,{l}_{L}^{2},\,{l}_{L}^{3},\,{l}_{L}^{4}\}$. Then a linear phase gradient of $2\pi x/{\rm{\Lambda }}$ (Λ = 1.6 µm) is imposed to the caustic phase function *P*_*L*_, so that the converted LCP beam will propagate with a deflection angle of $\alpha =\arctan (\lambda /{\rm{\Lambda }})$ and the total complex amplitude is $\exp [i({P}_{L}+2\pi x/{\rm{\Lambda }})]$. Second, for the RCP component ${P}_{R}=kQ+{L}_{R}(z)\phi$, and *L*_*R*_(*z*) = $\{{l}_{R}^{1},\,{l}_{R}^{2},\,{l}_{R}^{3},\,{l}_{R}^{4}\}$. A linear phase gradient $2\pi x/{\rm{\Lambda }}$ is also imposed to *P*_*R*_. Since the geometric phase shift is negative for the RCP component, the total complex amplitude for the converted RCP beam is $\exp [\,-\,i({P}_{R}+2\pi x/{\rm{\Lambda }})]$. Finally, the total geometric phase profile for producing space-variant vector vortex beam is obtained by superimposing the complex amplitudes of both RCP and LCP components and then calculating the argument of the summation complex function, as shown in Fig. 2(c). The total geometric phase profile from the superposition of the RCP component with helical phase charge of *L*_R_ and the LCP component with charge of *L*_L_ is then expressed as^15,55^:

7 $${\phi }_{geom}(x,y)=\text{arg}\{\exp [-\,i({P}_{R}+{{\rm{\Theta }}}_{z}+\frac{2\pi x}{{\rm{\Lambda }}})]+\exp [i({P}_{L}-{{\rm{\Theta }}}_{z}+\frac{2\pi x}{{\rm{\Lambda }}})]\},$$

where Θ_z_ is the phase difference between the RCP and LCP components as a function of propagation distance, and Θ_z_ has different values on different divided circular zones on the input plane. Θ_z_ determines the initial polarization orientation of vector vortex beam, with Θ_z_ = 0 and Θ_z_ = π/2 correspond to TM and TE polarization mode, respectively.

There are two channels of the vector vortex beams generated by the total geometric phase profile from Eq. (7). The first term of Eq. (7) with phase gradient 2*πx*/Λ will deflect the RCP component at a positive angle, while the second term with phase gradient −2*πx*/Λ will deflect the RCP component at a negative angle. For the LCP component, the deflection angle is reversed. For the channel at the positive angle, the beam is the superposition of $|R,\,{L}_{R}\rangle +|L,\,{L}_{L}\rangle$, while for the other channel at the negative angle, the beam is the superposition of $|R,\,-\,{L}_{L}\rangle +|L,\,-\,{L}_{R}\rangle$. Both channels produce vector vortex beams, and here only the channel at the positive angle is measured under the incident horizontal linear polarization, as shown in Fig. 2(d). In this work, three different categories of space-variant vector vortex beams are designed: (1) variation of polarization mode in the order of (TE → TM → TE → TM) with *m* = 1, 2 and *n* = 0; (2) variation of OAM value in the sequence of *n* = (1 → 2 → 3 → 4) with *m* = 1; (3) variation of TC of polarization state in the order of *m* = (0 → 1 → 2 → 3) with *n* = 4.

### Spatial variation of vector vortex beams

**Figure 3:**
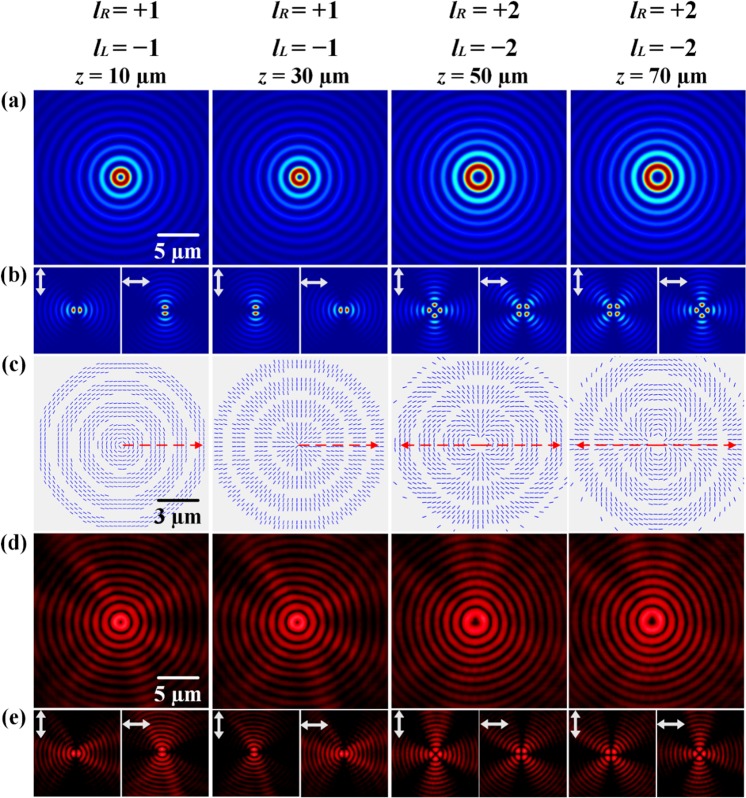
(**a**) The first category of vector vortex beam with the variation of polarization mode in the order of (TE → TM → TE → TM) with *m* = {1, 1, 2, 2} and *n* = 0. (**a**) Simulated beam intensity distributions without polarization analyzer at different propagation distances. (**b**) The corresponding intensity distributions analyzed with vertical or horizontal linear polarizer. (**c**) Simulated polarization distributions at different propagation distances. (**d**,**e**) The corresponding measured beam intensity distributions. The detailed transition processes of both beam intensity and polarization distributions are shown in the supplementary video.

The first type of vector vortex beam with the variation of polarization mode has *L*_*R*_(*z*) = {1, 1, 2, 2} and *L*_*L*_(*z*) = {−1, −1, −2, −2}, so that the OAM value *n* = (*L*_*R*_ + *L*_*L*_)/2 = 0 keeps invariant and the TCs of polarization state changes in the order of *m* = (*L*_*R*_ *−L*_*L*_)/2 = {1, 1, 2, 2}. The initial polarization orientation changes in the order of Θ_z_ = {π/2, 0, π/2, 0}, so the variation of polarization mode is (TE → TM → TE → TM). Figure 3(a) plots the simulated beam intensity distributions at different propagation distances. Figure 3(b) is the corresponding intensity distributions analyzed with vertical or horizontal linear polarizer, showing petal patterns with the variation of petal numbers 2 *m* = {2, 2, 4, 4}. Figure 3(c) gives the calculated polarization distributions. It can be seen from Fig. 3(b) at *z* = 10 µm, a dark line parallel to the transmission axis of the polarization analyzer is observed with the petal number of 2. The polarization distribution at *z* = 10 µm in Fig. 3(c) also clearly shows the initial polarization orientation of π/2 and the polarization rotation of 2π per round trip, indicating the azimuthally polarized vector beam with the TC of polarization state of 1 and TE polarization mode. At *z* = 30 µm, the dark line is perpendicular to the transmission axis of the analyzer, and the polarization distributions show the initial polarization orientation of 0 and the polarization rotation of 2π per round trip, giving the radially polarized vector beam with the TC of polarization state of 1 and TM polarization mode. At *z* = 50 µm and *z* = 70 µm, the beam spot becomes larger due to the increased vortex charge of $|{l}_{R,L}^{3,4}|$ = 2. In these two cases, the polarization rotation is 4π per round trip, so that the TC of polarization state equals 2 and the corresponding petal number becomes 4. Besides, the initial polarization orientation is π/2 for *z* = 50 µm and 0 for *z* = 70 µm, indicating the polarization mode is TE for *z* = 50 µm and TM for *z* = 70 µm, respectively. The evolution of vector vortex beam during the transition process is measured at the wavelength of 633 nm. Figure 3(d) displays the measured beam intensity distributions without polarization analyzer at different propagation distances, and Fig. 3(e) plots the corresponding intensity distributions analyzed with vertical or horizontal linear polarizer, which shows coincidence with the simulation results.

Furthermore, the transition process of the first-type vector vortex beam with the spatial variation of polarization mode in the order of (TE → TM → TE → TM) is demonstrated in the supplementary video, which shows the transition processes of both beam intensity and polarization distributions from *z* = 10 µm to *z* = 70 µm with the interval of 2 µm. It is shown that for the vector vortex beam, the original linear polarization (LP) state becomes the circular polarization (CP) state, and then changes into another LP state with different polarization orientation compared to the original LP state.

**Figure 4:**
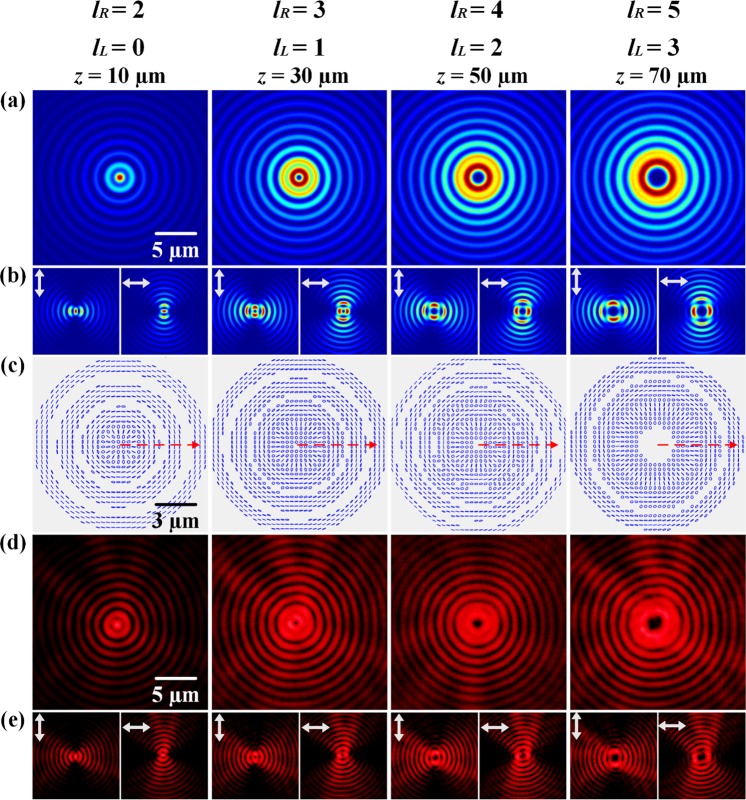
The second category of vector vortex beam with the variation of OAM value in the sequence of *n* = (1 → 2 → 3 → 4) with *m* = 1. (**a**) Simulated beam intensity distributions without polarization analyzer at different propagation distances. (**b**) The corresponding intensity distributions analyzed with vertical or horizontal linear polarizer. (**c**) Simulated polarization distributions. (**d**,**e**) Measured beam intensity distributions.

The second category of vector vortex beam with the variation of OAM value has *L*_*R*_(*z*) = {2, 3, 4, 5} and *L*_*L*_(*z*) = {0, 1, 2, 3}, giving the OAM value varies in the order of *n* = (*L*_*R*_ + *L*_*L*_)/2 = {1, 2, 3, 4} and the TC of polarization *m* = (*L*_*R*_ − *L*_*L*_)/2 = 1 keeps invariant. All these vector vortices are TE polarization modes with the initial polarization orientation Θ = π/2. The simulated beam intensity and polarization distributions are shown in Fig. 4(a–c). It is observed that the beam spot in Fig. 4(a) has the enlarged spot size as the OAM value *n* increases with respect to the propagation distance *z*. The petal structures in Fig. 4(b) always display the petal number of 2 at different propagation distances, due to the TC of polarization *m* = 1. From the polarization distributions in Fig. 4(c), it is observed that near the beam center the polarization state is circular polarization because in this case the superimposed Bessel beams have different orders. For the vortex helical phase of exp(*ilφ*), the beam field has a Bessel distribution with order *l* as ${J}_{l}(k{r}_{0}\rho /z)$. For the superposition of two vortex beams with exp(*il*_*R*_*φ*) and exp(*il*_*L*_*φ*) and *l*_*R*_ > *l*_*L*_, the Bessel function with order *l*_*R*_ exhibits a larger radius than that with order *l*_*L*_, so near the beam center, the intensity of LCP component is stronger than the intensity of RCP component, resulting in almost circular polarization near the beam center. Away from the beam center, the superposition of LCP and RCP components with the similar intensity exhibits nearly linear polarization. The initial polarization orientation is *π*/2, giving the TE polarization mode. The measured beam intensity distributions are shown in the Fig. 4(d,e).

**Figure 5:**
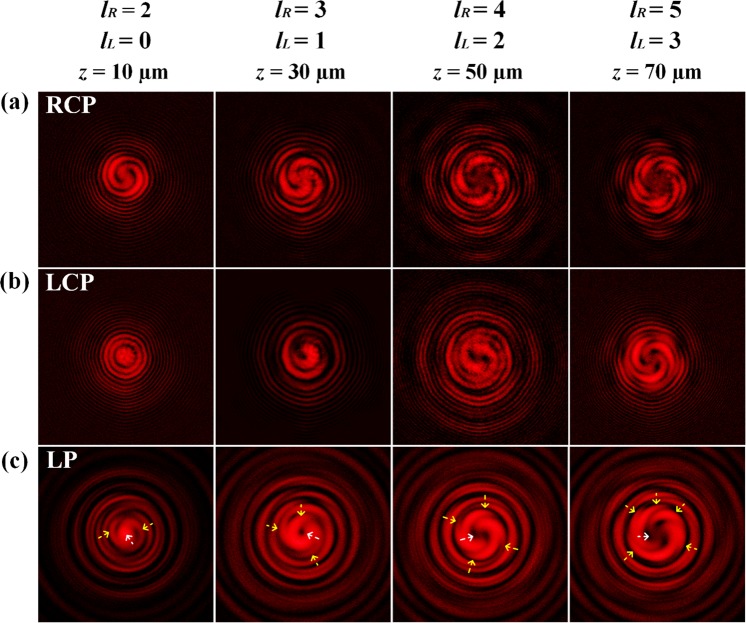
Measured interference patterns for the vector vortex beams with the reference spherical wave under (**a**) RCP, (**b**) LCP and (**c**) LP at different propagation distances. The interference structures at the beam center are marked by white arrows and the structures at the outer region are marked by yellow arrows.

In order to investigate the transition process of OAM value, the vector vortex beam is interfered with a reference spherical wave under RCP, LCP and LP at different propagation distances, as shown in Fig. 5. The vector beam is consisted of RCP and LCP components, and when the reference spherical wave has only RCP or LCP polarization, the interference results will only give the OAM information of the RCP or LCP component. If the reference spherical wave has linear polarization, due to the linear polarization is the superposition of RCP and LCP components, the interference with LP spherical wave will give the information of both RCP and LCP components in the vector beam. Here the RCP interference patterns in Fig. 5(a) only give the information of RCP components, showing a vortex spiral structure with the spiral number equals to *l*_*R*_. The LCP interference patterns in Fig. 5(b) only give the information of LCP components, showing a vortex spiral structure with spiral number equals to *l*_*L*_. The LP interference patterns in Fig. 5(c) contain both the inner part with *l*_*L*_ spirals (marked by the white arrow) and the outer part with *l*_*R*_ spirals (marked by the yellow arrows), giving the information of both RCP and LCP components in the vector vortex beam.

**Figure 6:**
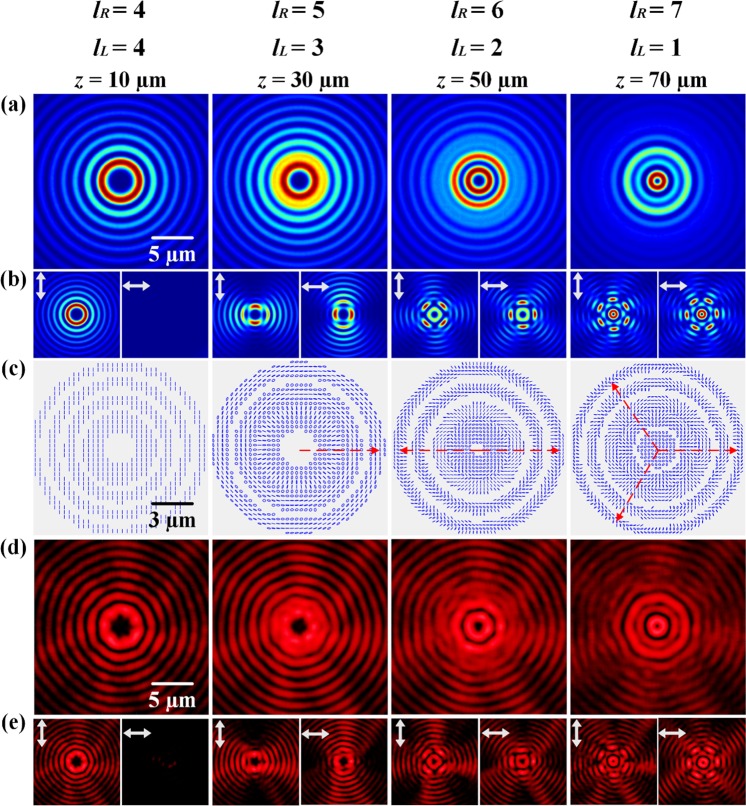
The third category of vector vortex beam with the variation of TC of polarization state in the order of *m* = (0 → 1 → 2 → 3) with *n* = 4. (**a**) Simulated beam intensity distributions without polarization analyzer at different propagation distances. (**b**) The corresponding intensity distributions analyzed with vertical or horizontal linear polarizer. (**c**) Simulated polarization distributions. (**d**,**e**) Measured beam intensity distributions.

The last category of vector vortex beam with the variation of TC of polarization state has the increased parameter *m* but the constant OAM value, where *L*_*R*_(*z*) = {4, 5, 6, 7} and *L*_*L*_(*z*) = {4, 3, 2, 1}, so that the OAM value is *n* = (*L*_*R*_ + *L*_*L*_)/2 = 4, and the TCs of polarization state changes in the order of *m* = (*L*_*R*_ − *L*_*L*_)/2 = {0, 1, 2, 3}. The simulated intensity and polarization distributions are shown in Fig. 6(a–c). The intensity distributions show the petal patterns with petal number of 2 *m*. At *z* = 10 µm, the beam is linearly polarized and there is no petal structure with *m* = 0, while at *z* = 30 µm, 50 µm and 70 µm, the corresponding petal numbers are 2 *m* = {2, 4, 6}. For the polarization distributions shown in Fig. 6(c), at *z* = 10 µm, the beam only contains vertical linear polarization state. At other propagation distances, the superposition of RCP and LCP components with different orders of Bessel functions results in circular polarization state near the beam center and linear polarization state with rotation of 2π∙*m* per round trip out of the beam center. Figure 6(d,e) are the corresponding measured beam intensity distributions, which agree with the simulation prediction.

## Discussion

In summary, the spatial variation of vector vortex beams with arbitrary polarization states and OAM values along the beam propagation has been demonstrated by using single plasmonic metasurfaces with the encoded initial phase profiles determined from the caustic theory. Based on the geometric phase profile induced by nanoslit antenna array, the deflected vector vortex beam is produced by the superposition of RCP and LCP component vortices with different helical phase charges transmitted through the same metasurface. Three different categories of vector vortex beams with the spatial variation of polarization mode, OAM value, and TC of polarization state are studied. Along the beam propagation distance, the detailed beam evolution process is analyzed for intensity profile, polarization distribution and OAM value. The demonstrated arbitrary spatial variation of vector vortex beam will advance many promising applications in spin-to-orbital angular momentum conversion, spin-orbit hybrid entanglement, quantum information processing, particle manipulation and transportation, and optical communication.

## Methods

### Simulations

The finite-integration time-domain (FITD) solver of the CST Microwave Studio software is employed to simulate the optical field distribution and the transmission spectrum. In the simulation, periodic boundary conditions are used along both *x* and *y* directions in the unit cell. The permittivity of gold is taken from spectroscopic ellipsometry data, and the refractive index of glass substrate is 1.45. The evolution processes of intensity and polarization distributions for vector vortex beams shown in Figs 3, 4 and 6 are calculated by using the Fresnel-Kirchhoff diffraction integral:

8 $${\rm{\Psi }}(x,\,y,\,z)=\frac{1}{i\lambda }{\iint }_{S}{\rm{\Psi }}({x}_{0},\,{y}_{0})[\frac{\cos (\overrightarrow{{\bf{n}}},{\bf{r}})-\,\cos (\overrightarrow{{\bf{n}}},{\bf{r}}{\boldsymbol{^{\prime} }})}{2}]\frac{{e}^{ikr}}{r}dS$$

where ${\rm{\Psi }}({x}_{0},{y}_{0})$ is the complex amplitude distribution located at the *z* = 0 plane with surface area *S* and normal direction $\overrightarrow{n}$, **r**′ is the vector between the source point and a point in the *z* = 0 plane, **r** is the vector between the point in *z* = 0 plane and a point in the plane at the propagation distance *z*, and *k* = 2π/*λ* is the wavevector. The polarization distributions are plotted by using the Stokes parameters.

### Sample fabrication

A 50 nm-thick gold film is deposited on a glass substrate using electron-beam evaporation. Then the nanoslit antenna arrays are milled in the gold film using focused ion beam system (FEI Helios Nanolab 600, 30 kV, 9.7 pA). The metasurface contains 300 × 300 unit cells, and each unit cell has the period of 330 nm and contains a nanoslit with size of 200 nm × 6  nm at a specified orientation angle which is determined by the designed geometric phase profile.

### Optical characterization

The transmission spectra of the metasurface under circular polarization basis shown in Fig. 1(d) are measured with a collimated broadband Tungsten-Halogen source, where a combination of a linear polarizer and a quarter-wave plate are used to convert the incident light to circularly polarized wave. The light beam is focused normally onto the sample using a 50× objective lens and the transmitted light is collected by another 10× objective lens to a spectrometer (Horiba, iHR 550). The deflected vector vortex beam is measured by a microscope imaging system with a 10× objective lens, a 0.5× tube lens and a CCD camera placed on a translation stage with a tilted angle of *α* = arctan(*λ*/Λ) = 21° to the optical axis, as shown in Fig. 2(d).

## Supplementary information

Supplementary video
